# Dysentery and Its Treatment

**Published:** 1887-10

**Authors:** Ad. Lippe

**Affiliations:** Philadelphia


### DYSENTERY AND ITS TREATMENT.

AD. LIPPE, M. D., PHILADELPHIA.

There is not the slightest doubt that our scientific knowledge is largely increased by comparisons. The illustrations here offered show how these comparisons may be utilized.

Purely homœopathic treatment is much facilitated by the little work of Drs. James B. Bell and W. T. Laird, used by all who are firm believers in the never-failing correctness of the Law of the Similars. It is an indispensable assistance in finding the similar remedy for the various diseases of the bowels. The faithful homœopathist adhering to the Law of the Similars will always cure his cases.

There is a very large number of medical men who *trade on a name*, and we will first give a few illustrations of their doings. A lady called on one of these nice young doctors, a graduate of the New York Homœopathic College. She had dysentery. The learned *trader on a name* gave her two bottles, and when asked, Why two remedies for one disease? this scientific individual explained himself, saying, one remedy is for the pain, the other is for the dysentery; take them in alternation. Did she? No. The lady had read the *Organon*, the *Chronic Diseases* by Hahnemann, *and* the article on Homœopathy by H. H. Furness, Esq., published in the *Encyclopedia Americana*, approved of and indorsed by the American Institute of Homœopathy. She evidently was better read than the New York graduate, and did *not* take the two remedies in alternation. She waited for a remedy from a homœopath and speedily recovered. Would it not have been more wise if this young Æsculapius had read the *Organon?* He would not then have been so silly as to give one remedy for the pain and the other for the dysentery.

A few weeks afterward this *trader on a name* was utterly outdone by a doctor who also *traded on a name.* At the most fashionable summer resort of this country an old homœopathic patient, a well-read lady, who for more than a quarter of a century had observed the results of strictly homœopathic treatment, was taken ill with dysentery, and called in a so-called homœopathic physician. He entered the sick-room with a "Saratoga trunk" filled with vials. First prescription, Arsenic$^{3}$, in water solution, and because the patient had much pain he added to the Arsenic solution just a little Morphia. The patient, of course, grew worse, and he then resorted to Coloc., Merc., etc., but added just a little Morphia to every solution. The patient grew worse rapidly; restless, sleepless nights; and, already scared by the Saratoga trunk, a true homœopath was summoned, and found on his arrival all secretions stopped, the patient desperately ill with brain symptoms. One dose of Aloe high relieved the abdominal pains, and the dysentery returned in full force. A few doses of Mercurius (high) controlled the case promptly. The first medical man, with the Saratoga trunk, *professes* to be a homœopath, but we declare that he is a vile eclectic, ignorant of Homœopathy, and evidently *trading on a name.* These men must be exposed without mercy, as they inflict terrible harm under the guise of the homœopathic name. That man surely never read the *Organon,* and if he did he could not take it in, but he seeks *recognition.*

We have shown what the *traders on a name,* who are also seeking recognition, do, what Homœopathy does, and now let us see what "the regulars" do. We give now what Fordyce says:

TREATMENT OF ACUTE DYSENTERY.—The inflamed edematous condition of the mucous membrane, and subsequent obstruction to passages from the bowel, is well known. Remembering the relief often obtained from the application of hot water to inflamed surfaces, and the beneficial effects of solutions of Bichloride of Mercury upon ulcerated surfaces, particularly of mucous membrane, the thought occurred to me that if I could carry the hot water into the bowel, allowing it to flow back in sufficient quantity to remove for a distance from above all fecal and offending matter, I should accomplish two important objects—*i. e.*, relief of pain, and prevent the absorption of poisonous matter up into the blood, the antiseptic properties of the Bichloride aiding in this latter object. Upon this I acted, using a soft rubber tube attached to a Davidson's syringe, passed carefully through this sensitive inflamed tissue, so as to carry the liquid above the rectum into the colon. The patient was placed on his side, with an oil-cloth beneath him, and four or five quarts of water as hot as could be borne were injected and allowed to flow back with whatever substance had accumulated or remained in the bowel above. When the water returned clear, then a quart or more of the solution of Bichloride, about 1 to 10,000, was injected and allowed to return in the same manner. The effect was immediate relief of pain and tenesmus. A suppository of Opium, one grain, was given and retained, and, for the first time after the attack, the patient slept seven hours, awakened refreshed, could take some food, and, if perfectly still, was free from pain. In about twelve hours slight return of pain was felt, and the same treatment repeated. This treatment was continued, with the Bichloride solution, four times, and the hot water alone was used for four or five days more, with suppositories of one grain of Opium, morning and night, with perfect recovery, no medicine being administered by the stomach, except one-grain doses of Quinine three times per day.—*Dr. Fordyce, Buff. Med. and Surg. Journal.*

Here we find an *accidental* cure, for no doubt the Bichloride of Mercury was homœopathic to the case. No medicine was given by the stomach except one-grain doses of Quinine three times per day. It is a pity, a great pity, that our scientific brethren of the allopathic school do not recognize such of our own brethren who, like the prevailing school of proclaimed healers, *trade on a name*—"healers" without a rudder or compass to show them the only true course for curing the sick.

